# Polymeric immunoglobulin receptor expression is correlated with poor prognosis in patients with osteosarcoma

**DOI:** 10.3892/mmr.2014.2110

**Published:** 2014-04-02

**Authors:** XUANWEI WANG, JINGYU DU, PENGCHENG GU, RILONG JIN, XIANGJIN LIN

**Affiliations:** Department of Orthopedics, the First Affiliated Hospital, Zhejianåg University School of Medicine, Hangzhou, Zhejiang 310003, P.R. China

**Keywords:** osteosarcoma, polymeric immunoglobulin receptor, prognosis

## Abstract

The prognosis of patients with osteosarcoma with distant metastasis and local recurrence remains poor. Increased expression of polymeric immunoglobulin receptor (pIgR) in tumor tissue has been detected in various types of cancer. However, the clinical significance of pIgR in osteosarcoma has yet to be elucidated. The present study aimed to investigate the prognostic value of pIgR in patients with osteosarcoma following surgical resection. pIgR expression was assessed using quantitative polymerase chain reaction analysis in cryopreserved osteosarcoma tissues from 22 patients, as well as using immunohistochemistry in paraffin-embedded osteosarcoma tissues from 136 patients. The association between pIgR expression, clinicopathological factors and long-term prognosis was retrospectively examined in these 136 patients. The prognostic significance of negative or positive pIgR expression in osteosarcoma was assessed using Kaplan-Meier survival analysis and log-rank tests. Univariate analysis indicated that patients with positive pIgR osteosarcoma tissue expression had a significantly worse overall survival (OS) compared with patients with negative pIgR osteosarcoma expression. Multivariate analysis revealed that positive pIgR expression in osteosarcoma tissues was an independent prognostic factor for OS following surgical resection (P<0.001). Furthermore, positive pIgR expression was significantly associated with poor prognosis in patients with osteosarcoma. These findings indicate that pIgR may be a novel predictor for poor prognosis in patients with osteosarcoma following surgical resection.

## Introduction

Osteosarcoma is the most common primary malignant bone tumor with highly malignant and invasive growth characteristics in adolescents and young adults (1). Osteosarcoma is associated with a poor prognosis, which is a result of its resistance to chemotherapy and tendency to metastasize to the lungs (2). Using traditional treatment methods, including chemotherapy, wide tumor resection and amputation, patients with osteosarcoma have a poor prognosis, with a five-year survival rate of <20% (3). The prognosis of patients with osteosarcoma has improved markedly, primarily due to the introduction of the extensive application of neoadjuvant chemotherapy and limb salvage surgery (4,5). However, the prognosis of patients with advanced osteosarcoma remains poor and advances in treatment are urgently required (6). Effective prognostic factors are important for clinicians to facilitate the selection of appropriate treatments for patients with osteosarcoma.

The polymeric (p) immunoglobulin (Ig) receptor (R) is a transporter of dimeric IgA and pentameric IgM, which are the first-line antibodies produced in response to infection. pIgR is widely expressed in epithelial cells and its expression is commonly increased by proinflammatory cytokines in response to viral or bacterial infection, linking innate and adaptive immunity (7–10). Upregulation of pIgR has been identified in colon cancer (11), breast cancer (12,13), endometrial carcinoma (14,15), bladder carcinoma (16) and hepatocellular carcinoma (HCC) (17,18). High levels of the cleaved extracellular domain of pIgR, designated as the secretory component, have also been detected in the sera of patients with lung (19,20) and pancreatic cancer (21), as well as patients exhibiting colon cancer with liver metastases (22). However, the clinical significance of pIgR in osteosarcoma has yet to be elucidated.

The present study aimed to investigate the association between pIgR expression and clinicopathological features. In addition, the potential of pIgR as a novel prognostic marker in patients with osteosarcoma following surgical resection was investigated.

## Materials and methods

### Patients and tumor tissue samples

Fresh tumor samples were obtained from 22 patients with osteosarcoma at initial surgery at the Department of Orthopedics, the First Affiliated Hospital, Zhejiang University School of Medicine (Hangzhou, China) between January 2010 and December 2012 for quantitative polymerase chain reaction (qPCR) analysis. Samples were snap-frozen and stored in liquid nitrogen until use. Patients had received no treatment prior to surgery. Paraffin-embedded osteosarcoma tissue samples were obtained from 136 patients undergoing surgical resection at the Department of Orthopedics, the First Affiliated Hospital, Zhejiang University School of Medicine between January 1998 and December 2007. None of the 136 patients had received chemotherapy or radiotherapy prior to resection. Following resection, patients were followed up every three months and the sections were reviewed by two pathologists to verify the histological assessment. Informed consent was obtained from all patients and the study protocol was approved by the Ethics Committee of the First Affiliated Hospital, Zhejiang University School of Medicine. The location of the tumors and distant metastases was determined using computed tomography (CT) and magnetic resonance imaging (MRI). The patients with osteosarcoma were staged according to the Enneking staging system (23). The staging workup involved CT scans of the chest to assess for pulmonary metastases, MRI and X-ray scans for local staging, and bone scans to assess for distant skeletal metastases. Patients exhibiting secondary malignancies, for which they had received prior chemoradiotherapy or surgery, or patients with pulmonary or nonpulmonary distant metastases on presentation to the First Affiliated Hospital, Zhejiang University School of Medicine, were excluded from the present study.

### qPCR analysis

Total RNA was extracted from the frozen tumor tissues using TRIzol^®^ Reagent according to the manufacturer’s instructions (Invitrogen Life Technologies, Carlsbad, CA, USA). Total RNA was reverse transcribed into single stranded complementary (c)DNA using a moloney-murine leukemia virus (M-MLV) reverse transcriptase (Promega Corporation, Madison, WI, USA). Briefly, RNA was denatured by heating for 5 min at 70°C, followed by rapid cooling on ice. The RNA was used for reverse transcription in a 25-μl reaction volume containing 2 μg total RNA, 25 units RNase inhibitor, 0.5 mmol/l each deoxyribonucleotide triphosphate, 1.5 μmol/l reverse primer and 200 units M-MLV reverse transcriptase. For reverse transcription, the reactions were incubated at 42°C for 60 min. The expression of pIgR was analyzed using a fluorescence-based real-time detection method with the ABI PRISM 7700 Sequence Detection System (PerkinElmer, Inc., Waltham, MA, USA) as described previously (24,25). The specific primer pairs and fluorescent probes for pIgR and glyceraldehyde-3-phosphate dehydrogenase (GAPDH) are shown in Table I. GAPDH served as an endogenous control. qPCR analysis was performed in triplicate for each sample. The 25-μl qPCR reaction consisted of 1 μl cDNA template, 1 μl each of sense and anti-sense primers, 0.75 μl 5′ FAM- and 3′ TAMARA-labeled oligonucleotide probes, 2 μl dNTP mixture, 5 μl 5X reaction buffer and 0.125 μl Taq DNA polymerase. The cycling conditions were as follows: 50°C for 2 min and 95°C for 10 min, followed by 46 cycles of 95°C for 15 sec and 60°C for 1 min. To determine the relative expression of pIgR mRNA in the individual tissue samples the Ct values were normalized using the Ct value for GAPDH mRNA (25).

### Immunohistochemistry

Selected tumor samples were fixed in 10% neutral-buffered formalin and embedded in paraffin. Sections (size, 5 μM) were cut, dewaxed, rehydrated and subjected to antigen retrieval. Subsequent to blocking endogenous peroxidase activity, the sections were incubated with the primary antibodies against pIgR (1:100; Epitomics Inc., Burlingame, CA, USA) overnight at 4°C. Immunohistochemistry was performed using the streptavidin-biotin-peroxidase complex method (Lab Vision Corporation, Fremont, CA, USA). The slides were analyzed and images were captured using an Olympus BX60 microscope (Olympus Corporation, Tokyo, Japan). Sections that are known to stain positively were incubated in each batch and negative controls were also established by replacing the primary antibody with pre-immune serum.

Expression analysis of pIgR in the tumor tissue was performed by comparing the staining intensity with the percentage of immunoreactive cells. Staining intensity was arbitrarily scored on a scale of four grades: 0, No staining; 1, weak staining; 2, moderate staining; and 3, strong staining. The percentage of positive cells was scored according to the following grades: 0, 0%; 1, 1–25%; 2, 26–50%; and 3, >50%. pIgR staining positivity was determined using the following formula: Overall score = positive percentage score × staining intensity score. A score of 0 was termed 0, a score >0 and ≤2 was termed 1, a score >2 and ≤6 was termed 2 and a score >6 and ≤9 was termed 3. Tumor samples graded as level 0 or 1 were defined as negative for pIgR expression, whereas samples graded as level 2 or 3 were defined as positive for pIgR expression.

### Follow-up

Patient follow-up consisted of physical examination, including CT, MRI and X-ray scans every three months for the first five years, then annually thereafter. Patients were followed up until mortality or until the date of the final follow-up. Follow-up was terminated on December 31, 2012. The median follow-up was 41.7 months (range, 10–179 months).

### Statistical analysis

All statistical analyses were performed using SPSS 16.0 software (SPSS, Inc., Chicago, IL, USA). Data are expressed as the mean ± standard error of the mean. Clinicopathological parameters were analyzed via two-tailed χ^2^ and two-tailed t-tests to assess the association between pIgR expression and clinicopathological parameters. Overall survival (OS) curves for patients with positive and negative pIgR expression were estimated using the Kaplan-Meier method. Survival functions were compared using the log-rank test. Univariate and multivariate analyses were based on the Cox proportional-hazards regression model. Factors that significantly influenced OS were used in the Cox proportional-hazards regression model for multivariate analysis. P<0.05 was considered to indicate a statistically significant difference.

## Results

### pIgR expression in osteosarcoma

qPCR analysis was performed to assess *pIgR* gene expression in 22 fresh frozen osteosarcoma samples. The housekeeping gene, *GAPDH* served as a control. pIgR expression was found to be positive in 15/22 (68.2%) patients with osteosarcoma (Table II).

**Figure 1:**
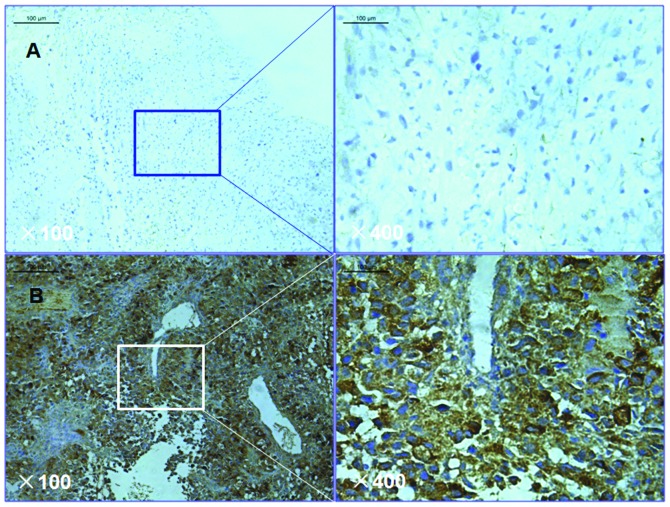
Polymeric immunoglobulin receptor (pIgR) expression in osteosarcoma tissues visualized via immunohistochemical staining. (A) Negative and (B) positive expression of pIgR. (A&B) Left panel; magnification, ×100: Right panel, magnification, ×400.

To determine the frequency of positive expression of the *pIgR* gene in osteosarcoma, pIgR expression was analyzed in 136 paraffin-embedded osteosarcoma tissue samples using immunohistochemical staining. Among the 136 osteosarcoma samples, *pIgR* was observed to be expressed in 93/136 (68.4%) samples (Fig. 1). This finding indicates that pIgR may be key in osteosarcoma. Table III demonstrates the association between pIgR expression and clinicopathological characteristics of the 136 osteosarcoma tissue samples, including age, gender, tumor location, histological type and grade.

### pIgR expression is associated with poor survival in patients with osteosarcoma

**Figure 2:**
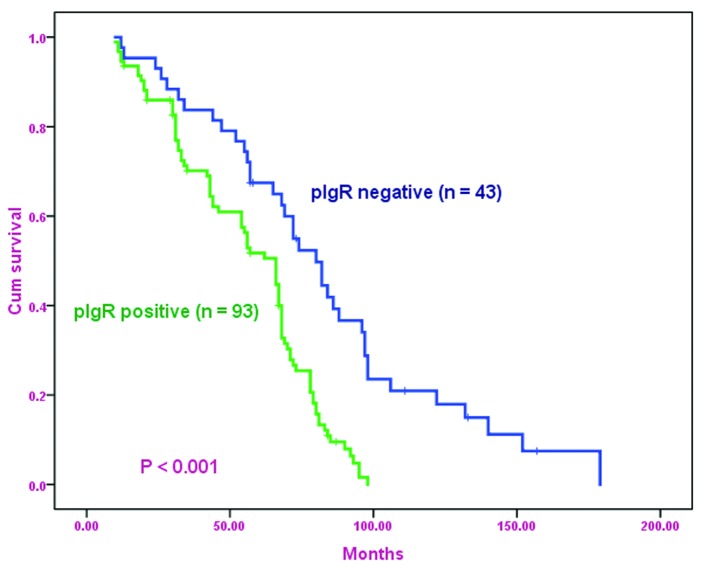
Kaplan-Meier survival curves of patients with osteosarcoma undergoing surgical resection, grouped by pIgR expression in tumor tissues. The survival rate of the patients with osteosarcoma in the pIgR-negative expression group (n=43) was significantly higher than that of the patients in the pIgR-positive expression group (n=93). P<0.001. pIgR, polymeric immunoglobulin receptor; Cum, cumulative.

The OS curves for patients with osteosarcoma, subdivided on the basis of pIgR expression, are shown in Fig. 2. Positive pIgR expression was found to be associated with poor prognosis in patients with osteosarcoma (log-rank test, P<0.001). Univariate analysis revealed that patients who exhibited a positive expression for pIgR had a significantly poorer prognosis compared with those who exhibited a negative expression for pIgR (P<0.001; Table IV). Multivariate analysis demonstrated that positive pIgR expression was an independent and significant predictor in OS (Table V).

## Discussion

Osteosarcoma is the most common type of malignant primary bone tumor (1). Osteosarcoma has a high metastatic potential, most commonly spreading to the lungs and bone (26). The relatively high mortality rate associated with osteosarcoma is predominantly associated with systemic metastasis, particularly pulmonary metastasis (27). The five-year survival rate for patients with osteosarcoma metastases is 20% compared with 65% for patients with localized disease and the majority of the mortalities associated with osteosarcoma are the result of metastasis (5,28). Despite aggressive treatment modalities, including high-dose chemotherapy and wide tumor resection, the five-year survival rate for patients with osteosarcoma is between 55 and 60% and <40% for patients with pulmonary metastases (4,5). Thus, the identification of biomarkers, which offer prognostic insight and guide clinical treatment, is considered to be important.

The present study aimed to investigate the prognostic value of pIgR in patients with osteosarcoma following surgical resection. pIgR is a glycoprotein present on glandular epithelial cells that functions as a receptor for pIg. pIgR transports pIgA into external secretions as secretory IgA, which is critical for mucosal tissue defense (29). pIgR has been reported to be overexpressed in colon (11) and breast cancer (12,13), endometrial carcinoma (14,15), bladder carcinoma (16), and HCC (17,18); however, the clinical significance of pIgR remains unknown. The prognostic value of pIgR in patients with malignancy also remains unclear. Ai *et al* (18) were the first to report the clinical significance of pIgR in HCC. pIgR was identified as a prognostic biomarker for HCC and was shown to have a role in the hepatitis B infection, chronic liver inflammation, the induction of the epithelial-mesenchymal transition, HCC recurrence and metastatic progression (18). The role of pIgR in osteosarcoma required investigation, thus the present study aimed to immunohistochemically assess pIgR expression in 136 pretherapeutic tumor samples and correlate the expression with clinicopathological parameters in order to identify the potential prognostic implications of pIgR in osteosarcoma.

In the present study, pIgR expression was analyzed in cryopreserved osteosarcoma tissues from 22 patients using qPCR analysis and was found to be expressed in 15 (68.2%) patients. pIgR expression was subsequently assessed in paraffin-embedded osteosarcoma tissue samples from 136 osteosarcoma patients with clinical follow-up records; positive pIgR expression was identified in 93 (68.4%) of the paraffin-embedded osteosarcoma tissue samples. Univariate analysis revealed that OS for patients with a positive pIgR expression in osteosarcoma tissues was significantly poorer compared with patients with negative pIgR expression. Furthermore, multivariate analysis showed that positive pIgR expression in osteosarcoma tissues was an independent prognostic factor for OS following surgical resection (P<0.001). To the best of our knowledge, this is the first study to indicate that pIgR has a role in osteosarcoma, however, this requires further investigation.

In conclusion, to the best of our knowledge, this is the first study to show that positive expression of pIgR is significantly associated with a poor prognosis in osteosarcoma patients. Therefore, pIgR may be a novel predictor for poor prognosis in osteosarcoma patients following surgical resection and may be a promising candidate for targeted osteosarcoma therapy.

## Figures and Tables

**Table I:** Sequences of primers and probes used for quantitative polymerase chain reaction analysis.

Primer/probe	Sequence
Polymeric immunoglobulin receptor	
Forward primer	5′-CTCTCTGGAGGACCACCGT-3′
Reverse primer	5′-CAGCCGTGACATTCCCTG-3′
TaqMan probe	6FAM-5′-AGATCAAGATTATCGAAGGAGAACCAAACCTC-3′-TAMRA
Glyceraldehyde-3-phosphate dehydrogenase	
Forward primer	5′-TCCATGACAACTTTGGTATCGTG-3′
Reverse primer	5′-ACAGTCTTCTGGGTGGCAGTG-3′
TaqMan probe	6FAM-5′-AAGGACTCATGACCACAGTCCATGCCA-3′-TAMRA

**Table II:** pIgR mRNA expression in osteosarcoma samples.

	pIgR mRNA expression	
	
Tumor tissue	Samples (positive/total)	Level of expression (mean ± SEM)
Osteosarcoma	15/22	0.32±0.07

mRNA expression was assessed using quantitative polymerase chain reaction analysis and was expressed relative to GAPDH mRNA expression. The criterion for detectable expression for each transcript was an mRNA level ≥0.02% of GAPDH mRNA. pIgR, polymeric immunoglobulin receptor; SEM, standard error of the mean.

**Table III:** Association between pIgR expression and clinicopathological parameters in 136 patients with osteosarcoma.

	pIgR expression		
	
Parameter	Positive	Negative	P-value
Patients (n/%)	93/68.4	43/31.6	-
Age (years; mean ± SEM)	22.7±7.2	24.3±8.5	0.832
Gender			
Female (n/%)	43/46.2	21/48.8	0.637
Male (n/%)	50/53.8	22/51.2	-
Tumor location			
Femur	45/48.4	22/51.2	0.635
Tibia	23/24.7	11/25.6	-
Humerus	8/8.6	3/7.0	-
Fibula	6/6.5	2/4.7	-
Pelvis	5/5.4	2/4.7	-
Other	6/6.5	3/7.0	-
Histological type			
Osteoblastic	47/50.5	23/53.5	0.712
Chondroblastic	22/23.7	11/25.6	-
Fibroblastic	18/19.4	7/16.3	-
Telangetatic	6/6.5	2/4.7	-
Histological grade			
Low	22/23.7	9/20.9	0.563
High	71/76.3	34/79.1	-

pIgR, polymeric immunoglobulin receptor; SEM, standard error of the mean.

**Table IV:** Univariate analysis of OS in patients with osteosarcoma following surgical resection.

	OS	
	
pIgR expression	Patients (n)	P-value
Positive	93	<0.001
Negative	43	-
Total	136	-

pIgR, polymeric immunoglobulin receptor; OS, overall survival. P<0.001, compared with the patients with negative pIgR expression.

**Table V:** Multivariate analysis of overall survival in patients with osteosarcoma following surgical resection.

Parameter	HR (95% CI)	P-value
Positive pIgR expression	2.582 (1.763–3.585)	<0.001

HR, hazard ratio. CI, confidence interval; pIgR, polymeric immunoglobulin receptor.
